# An Allelochemical from *Myrica gale* with Strong Phytotoxic Activity against Highly Invasive *Fallopia* x *bohemica* Taxa

**DOI:** 10.3390/molecules16032323

**Published:** 2011-03-10

**Authors:** Jean Popovici, Cedric Bertrand, Dominique Jacquemoud, Floriant Bellvert, Maria P. Fernandez, Gilles Comte, Florence Piola

**Affiliations:** 1 Universite de Lyon, F-69622 Lyon, France, and Universite Lyon 1, Villeurbanne, CNRS, UMR5557, Ecologie Microbienne, 43 Boulevard du 11 Novembre 1918, F-69622 Villeurbanne Cedex, France; E-Mails: dominique.jacquemoud@uni-jena.de (D.J.); floriant.bellvert@univ-lyon1.fr (F.B.); fernandz@biomserv.univ-lyon1.fr (M.P.F.); gilles.comte@univ-lyon1.fr (G.C.); 2 Laboratoire de Chimie des Biomolécules et de l’Environnement, Université de Perpignan, F-66860, Perpignan, France; E-Mail: cedric.bertrand@univ-perp.fr; 3 Laboratoire d’Ecologie des Hydrosystèmes Fluviaux, Unité Mixte de Recherche 5023, Centre National de la Recherche Scientifique, Université Lyon 1, 43 Boulevard du 11 Novembre 1918, 69622 Villeurbanne Cedex, France; E-Mail: florence.piola@univ-lyon1.fr

**Keywords:** biological control, *Myrica gale*, *Fallopia*, allelopathy, dihydrochalcone, invasive plant

## Abstract

We report the identification of the allelochemical 3-(1-oxo-3-phenylpropyl)-1,1,5-trimethylcyclo-hexane-2,4,6-trione, known as myrigalone A, from the fruits and leaves of *Myrica gale*. The structure of the compound was confirmed by high-resolution techniques (UV, MS and NMR analysis). The compound is phytotoxic towards classical plant species used for allelochemical assays and also against *Fallopia* x *bohemica*, a highly invasive plant. Application of either powdered dry leaves or dry fruits of *M. gale* also showed *in vitro* phytotoxic activity. We hypothesize that *M. gale* could be used as a green allelopathic shield to control *Fallopia* x *bohemica* invasion, in addition to its potential use as an environmentally friendly herbicide.

## 1. Introduction

Biological invasions are recognized as a result of global change and are of growing interest in the biological sciences [1] because of their potential effects on biodiversity. Invaded ecosystems are generally considered as disrupted and the native species as strongly threatened. Many invasive plant species are known to affect plant and animal communities [2,3], ecosystem functioning [4,5,6], soil properties and nutrient fluxes [7,8] in their new environments. The impact of the invasion can be site-specific and is linked, among other factors, to soil properties in the novel habitat prior to the invasion. The effect of the invasion on the soil nutrients (increased or decreased) is dependent on the original soil chemical conditions, even if the plant can be invasive in a broad range of soil nutrient conditions [9].

Invasive knotweeds (species complex *Fallopia*) represent a particularly aggressive taxonomic group in Europe and Northern America [10,11,12]. The genus *Fallopia* (Polygonaceae) includes two herbaceous perennial Asian polyploid species (*F. japonica* and *F. sachalinensis*), that have now become widespread and highly invasive weeds. Recent hybridization events implicating *F. japonica* and *F. sachalinensis* in the invasion area have led to a new species being formed, *F*. x *bohemica*, or more precisely to a complex of polyploid hybrids [13].

Ecosystems invaded by Japanese knotweeds are generally considered as modified and the endemic biodiversity as threatened [14,15,16,17]. Many watercourses are now highly invaded, with large populations of *Fallopia* being found along riverbanks in countries such as the UK [18], the Czech Republic [19] and France (F. Piola personal observation and [20]). The invasive capacity of the *Fallopia* complex may be the result of exceptionally high growth rate, gigantism and extremely effective vegetative multiplication, as found for other invasive species [21]. The invasive potential of the *Fallopia* complex may also result from its more direct effects on the environment, through the production of substances toxic to organisms native to the invaded habitat. It is now known that chemical compounds possessing antifungal, antiherbivory and antimicrobial properties or with phytotoxic (allelopathic) effects confer some invasive plants benefits in new environment. Invasive plant strategies based on such compounds constitute the hypotheses named « Novel Weapons Hypothesis » or « Allelopathic Advantage against Resident Species (AARS) » [22,23,24,25]. Extracts of *F. japonica* and *F.* x *bohemica* also exhibit phytotoxic activities [26,27,28] and antifungal properties [29,30]. 

We hypothesize that the novel weapon theory could be applied to invasive species themselves: plants producing allelochemicals unknown to invasive plants could affect the growth of invasives and may provide a safe, novel method of biocontrol. The plant used as chemical weapon against the invasive species must not itself become an invasive plant in alien environments. Consequently, the “weapon plant” must have well-defined, specific and known ecological requirements restricting it from growing in most environments and ensuring it is unknown to the invasive plant. *Myrica gale* (*Myricaceae*) fulfils these conditions, growing only in acidic wet to flooded edaphic conditions [31]. This shrub is found in environments such as the wet and flooded area around lakes, along rivers or in peat bogs and has a wide distribution from Northern and Western Europe the American continent. The fruits and leaves of *M. gale* are covered in droplets made up of exuded secondary metabolites [32]. The composition of the fruit exudates is made of a high diversity of chemical structures, including essential oils and flavonoids from the class of dihydrochalcones and for which several biological activities have been reported [33,34,35]. Although phytotoxic activity has already been observed for other *Myricaceae* species [36], no allelopathic properties have ever been described for *M. gale*. Here, we investigated the phytotoxic activity of *M. gale* exudates against a variety of plants used in classical phytotoxic assays and against *F.* x *bohemica*. We also report the identification of the active compound responsible for phytotoxic activity. We finally discuss the implications of our results for the development of a successful biocontrol solution for *Fallopia* in France.

## 2. Results and Discussion

### 2.1. Allelopathic potential of M. gale fruit exudates against F. x bohemica

**Figure 1:**
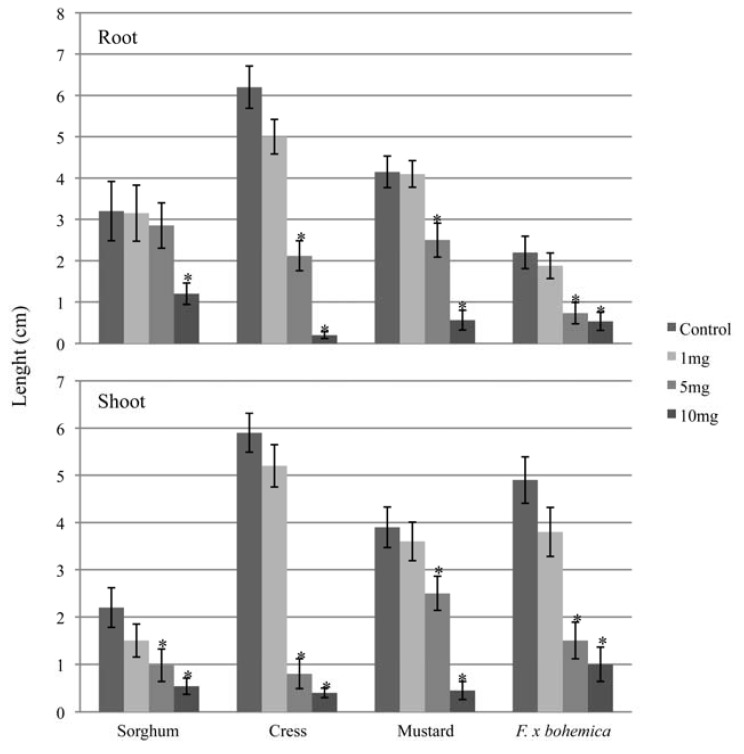
The effects of *M. gale* fruit exudate extracts on the root and shoot growths of sorghum, cress, mustard and *F.* x *bohemica*. Concentrations are the total quantities of dry exudate extracts added to each Petri dish for the bioassays. Means ± SE are shown for three independent experiments with 5–10 plants per treatment. Significant differences with the control treatment (*P* < 0.05, Tukey’s test) are indicated by *.

The methanol extract of *M. gale* fruit exudates inhibited the root and shoot growth of all the plant species tested, including *F.* x *bohemica* (Figure 1).

Root and shoot growth was inhibited for all the plant species when at least 5 mg of extract was applied, except for sorghum where root growth was significantly inhibited only when 10 mg of exudate was applied. Germination rates were also reduced by *M. gale* fruit exudate extracts (Table 1). 

Cress and mustard were the most affected, with only 1% and 24% of germinated seeds, respectively, when 10 mg of fruit exudate was applied. By contrast, in the control assays 100% and 93% of seeds germinated for cress and mustard, respectively. Germination rates of sorghum and *F.* x *bohemica* were also slightly reduced by *M. gale* fruit exudates but to a lesser extent (Table1).

The taxa of *Fallopia* are highly invasive plants threatening numerous ecosystems in Europe and North America. There is currently no successful strategy to eliminate *F.* x *bohemica* and effective control methods are urgently needed. In this work we have hypothesized that one of the mechanisms by which a plant becomes invasive, the Novel Weapons Hypothesis, might be used against invaders themselves. We show that indeed *M. gale* fruit extracts exhibit phytotoxic activity against *F.* x *bohemica*.

### 2.2. Identification of an allelochemical from M. gale fruit exudates

**Figure 2:**
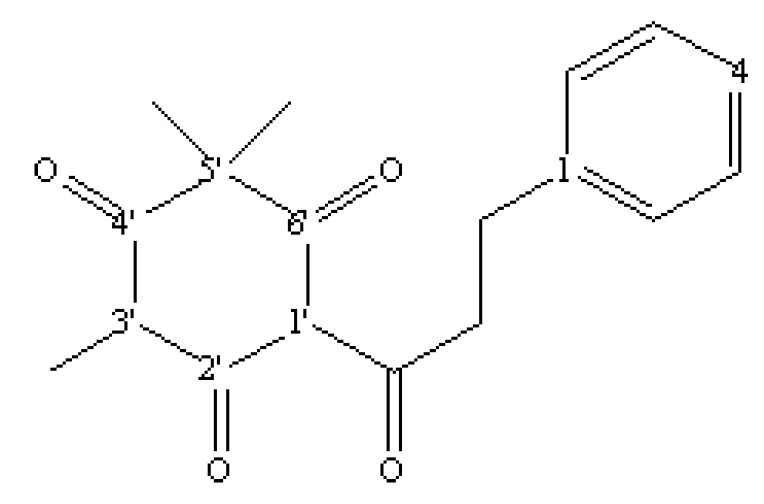
Chemical structure of 3-(1-oxo-3-phenylpropyl)-1,1,5-trimethylcyclohexane-2,4,6-trione (myrigalone A).

An active substance (9 g) was purified from *M. gale* fruit exudates (350 g of fruits). Based on a literature comparison of its ^1^H-, ^13^C-NMR, and MS data, as well as its UV spectra, the molecule was identified as 3-(1-oxo-3-phenylpropyl)-1,1,5-trimethylcyclohexane-2,4,6-trione, also known as myrigalone A (MyA), a flavonoid from the dihydrochalcone class (Figure 2). The compound has only been described from *M. gale* [34,37] but, to our knowledge, no phytotoxic activity has ever been reported for it. HPLC analysis revealed that MyA was also present in *M. gale* leaves (data not shown).

**Figure 3:**
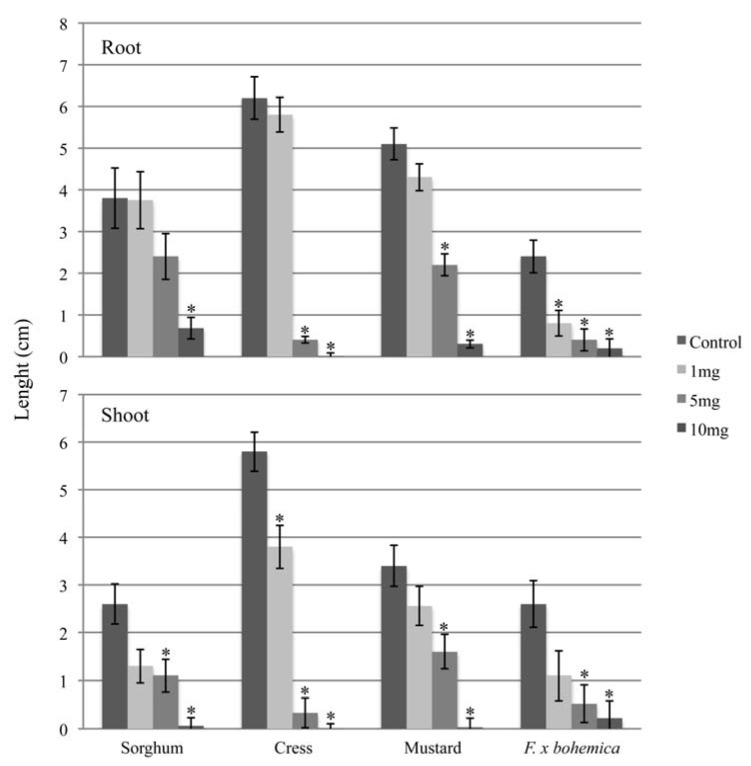
The effects of myrigalone A on the root and shoot growths of sorghum, cress, mustard and *F.* x *bohemica*. Concentrations are the total quantities of myrigalone A added to each Petri dish for the bioassays. Means ± SE are shown for three independent experiments with 5–10 plants per treatment. Significant differences with the control treatment (*P* < 0.05, Tukey’s test) are indicated by *.

We show that this compound is phytotoxic against plants generally used in allelochemical bioassays (cress, mustard and sorghum) as well as against *F.* x *bohemica* (Figure 3). 

This molecule is produced in a high yield in *M. gale* fruit exudates (40% of the total fruit exudates) and, to a lesser extent, in the leaves. Myrigalone A shares structural features with the natural β-triketone phytotoxin leptospermone, produced by the bottlebrush plant (*Callistemon* spp.). The β-triketone moiety is responsible for the phytotoxicity of the compound and its molecular target is *p*-hydroxyphenylpyruvate dioxygenase (HPPD), an enzyme involved in the biosynthesis of tocochromanols (tocopherols and tocotrienols) and prenylquinones [38,39,40]. Inhibition of HPPD causes photodynamic bleaching of the foliage. Triketones have mainly been described in *Myrtaceae* species such as *Leptospermum*, *Eucalyptus*, and *Corymbia* and commercial herbicides have been developed based on the triketone moiety (sulcotrione, mesotrione…).

The mechanisms by which the plant producing allelochemicals is protected from these compounds are still unclear. However it is thought that the site of production of the compounds, glandular trichomes and other glands, might compartmentalize the bioactive products and thus protecting the plant from autotoxic effects. Such glands can be found on *M. gale* leaves as well as in other plants producing allelochemicals such as *Myrtaceae* [32]. In *M. gale* fruits, myrigalone A is exuded outside the fruit, on the surface of the lignified drupe, possibly to protect the seed (unpublished data). 

### 2.3. Phytotoxicity of whole fruits and leaves of M. gale

**Figure 4:**
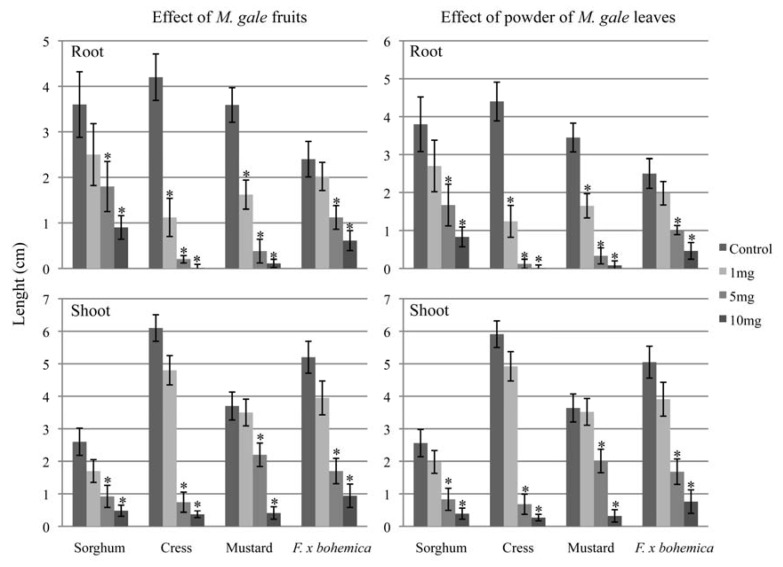
The effects of *M. gale* entire fruits and leaf powder on the root and shoot growth of sorghum, cress, mustard and *F.* x *bohemica*. Concentrations are the total quantities of fruits or leaf powder added to each Petri dish. Means ± SE are shown for three independent experiments, with 5–10 plants per treatment. Significant differences with the control treatment (*P* < 0.05, Tukey’s test) are indicated by *.

Entire dry fruits and powdered dry leaves of *M. gale* inhibit the root and shoot growth of all the tested plant species, similar to the inhibitory effects observed for methanol extracts (Figure 4). 

The results show root growth inhibition is observed in cress and mustard as soon as 100 mg of entire fruit or leaf powder is applied. For all the plants tested, root and shoot growths are inhibited when 200 mg of entire fruits and leaf powder is applied.

The results described in this paper show that both pure myrigalone A and fruit exudate methanol extract can inhibit the root and shoot growth of *F.* x *bohemica*. However, applying solvent-dissolved phytosanitary products in the field can be challenging and the toxicity of these solutions must be taken into account. 

In order to avoid the utilization of solvents for applying allelochemicals, we showed that the use of *M. gale* entire fruits or leaf powder sprinkled was enough to demonstrate a phytotoxic effect on the plant species tested, including *F.* x *bohemica*. Our results strengthen the argument for the development of allelopathic shields against alien species. Indeed, the identification of endemic allelopathic plants in an environment may lead to use these plants as a source of allelopathic compounds unknown to invading species. Such plants could also be used as green allelopathic barriers to prevent the invasion of a threatening species within their own environment. Consequently, one “weapon plant” will not be adapted to each environment and as such, for every threatened environment, the identification and use of a suitable allelopathic plant must be done. We are currently testing this hypothesis using *M. gale* to control *F.* x *bohemica* species in Rhône-Alpes region of France. *M. gale* appears to be suitable for testing the concept of green allelopathic shields because it grows only around wet and flooded areas as well as river shores, sites that are currently heavily invaded by *F.* x *bohemica* in the Rhône-Alpes. We acknowledge however that the implementation from laboratory studies to field experiment is a difficult and critical step and identifying other potential “weapon plant” might be necessary to fully achieve protection against *F* x *bohemica* in this environment. Moreover, even if seed germination appears to be a mechanism of dissemination of *F*. x *bohemica* [41], its propagation is mainly vegetative through rhizome fragments. We are currently evaluating *M. gale* allelopathic properties on rhizome fragments regeneration. Other experiments are currently undertaken to evaluate in glasshouse and mesocosm conditions the possible use of *M. gale* as an allelopathic shield. These include the evaluation of *M. gale* effects on other plant species living in the same ecosystem, even if since they have coevolved together, no phytotoxicity is expected to be observed. Finally those mesocosm experiments will provide data about *M. gale* and *F*. x *bohemica in situ* competition along with possible phytotoxic activity of *F*. x *bohemica* against *M. gale*. If the results of those experiments show that *M. gale* fulfils the requirements to be an allelopathic barrier, then implementation on invaded sites in the Rhône-Alpes region to evaluate this biocontrol strategy will be performed.

## 3. Experimental

### 3.1. General

Chromatographic analysis of extracts was achieved by HPLC on an Agilent 1200 series HPLC instrument equipped with a degasser (G132A), a quaternary pump module (G1311A), an automatic sampler (G1329A) and a DAD (DAD G1315B). Separations were carried out using a Kromasil RP18 column (250 × 4.6 mm, 5 µm, 100 Å) with a linear gradient of acetonitrile in water from 0% to 100% in 60 min supplemented with formic acid (0.4%). Chromatograms were recorded between 200 and 700 nm and a specific channel set at 280 nm was used to monitor chromatographic traces. Structural elucidation was achieved by UV spectroscopy (Agilent 8453 UV spectrophotometer) recorded between 220 and 500 nm, HPLC-MS and by ^1^H- and ^13^C- mono and bidimensional NMR (Bruker DRX 500). For MS analysis, the HPLC system described above was interfaced with an HP MSD 1100 series allowing the same chromatographic conditions as those used for HPLC-DAD analysis. The operating conditions of the mass spectrometer with an APCI interface were: gas temperature 330 °C at a flow rate of 9.0 L/min, nebulizer pressure 50 p.s.i, quadripole temperature 30 °C, capillary voltage 4,000 V and fragmentor 100. The full scan spectrum from m/z 100 to 900 in both positive and negative ion mode was recorded

### 3.2. Plant material

*M. gale* fruits and leaves were collected in December 2004 on the shore of Biscarosse Lake, Bordeaux, France. A voucher specimen was deposited at the Herbarium of the University Claude Bernard Lyon1, Villeurbanne, France, under the name ‘Collection Piola’ and collector number 3. Air-dried fruits (500 g) were sonicated (Branson 2510, 40KHz) twice in methanol (2L) and evaporated, allowing us to obtain 50 g of dried residue. Leaves were dried and ground and compounds were extracted by sonication in methanol. The two extracts were then diluted in methanol for the further experiments. *Fallopia* x *bohemica* akenes were collected in January 2008 on stands growing along the river Dorlay in the Loire region of France. The akenes were collected randomly on several shoots of *Fallopia,* pooled and stored at 4 °C.

### 3.3. Phytotoxicity assays 

Total fruit exudates and purified myrigalone A were first dissolved in methanol. Aliquots of the extracts were evaporated on filter paper (Whatman n°2) placed in a 9 cm-Petri dish to obtain final quantities of 1, 5 and 10 mg of dry extract per Petri dish. Three additional sheets of filter paper were placed underneath the sheet with the extracts and moistened with 8mL of water. Controls were obtained by placing methanol only on the paper sheet, which was then evaporated before adding water. Five, 10, 20 and five seeds of respectively sorghum (*Sorghum saccharatum*), mustard (*Sinapis alba*), cress (*Lepidium sativum*) and *F.* x *bohemica* were placed on the paper sheets in the Petri dishes and incubated for 72 h-10 days at 25 °C in darkness. After incubation, germination rate and shoot and root lengths were measured. Each bioassay was repeated three times. Significant differences between treatment and control plants were examined by Tukey’s test (R software 2.9.0).

A second bioassay protocol was developed to evaluate the phytotoxicity of whole *M. gale* organs (fruits and leaves) without methanol extraction. The protocol was similar to that described above with the key difference that test seeds were sprinkled with entire fruits or dry leaf powder instead of having methanolic extracts applied to them. One hundred, 200 and 300 mg of fruits or leaves were sprinkled on each Petri dish. After 72 h of growth, shoot and root lengths were measured and compared to non-sprinkled control plants.

### 3.4. Extraction and isolation

Gel filtration on Sephadex LH-20 (600 × 45 mm, methanol) of fruit exudate extract (35 g) resulted in 10 major fractions that were assessed for phytotoxic activity as described above. One fraction was active and its content was analysed by HPLC. This fraction, weighing 8 g, was composed of a single molecule. Structural elucidation was achieved by UV spectroscopy, Mass Spectrometry and NMR analyses and comparison with the literature led to the identification of myrigalone A [35,37].

## 4. Conclusions

We have shown that *M. gale* fruits and leaves extracts exhibit phytotoxic activities against different plant species, including against the invasive species *Fallopia*. The phytotoxicity is due to the presence of 3-(1-oxo-3-phenylpropyl)-1,1,5-trimethylcyclohexane-2,4,6-trione (myrigalone A), a dihydro-chalcone present in both the fruits and leaves. We hypothesize that *M. gale* could be used in the future as an allelopathic green shield to control invasion of *F.* x *bohemica* in some environments, in addition to being a potential source of environmentally friendly herbicides.

## Figures and Tables

**Table 1:** Effects of *M. gale* methanol fruit exudate extracts on the germination rates of sorghum, cress, mustard and *F.* x *bohemica*. Concentrations are the total quantities of dry exudate extracts added to each Petri dish. Thirty plants were used for each treatment.

Treatment	Sorghum	Cress	Mustard	*F.* x *bohemica*
**Control**	90%	100%	93%	88%
**1mg**	87%	100%	94%	90%
**5mg**	80%	7%	37%	70%
**10mg**	70%	1%	24%	70%
